# Pathogenesis of Sarcopenia in Chronic Kidney Disease—The Role of Inflammation, Metabolic Dysregulation, Gut Dysbiosis, and microRNA

**DOI:** 10.3390/ijms25158474

**Published:** 2024-08-03

**Authors:** Estera Bakinowska, Joanna Olejnik-Wojciechowska, Kajetan Kiełbowski, Anastasiia Skoryk, Andrzej Pawlik

**Affiliations:** 1 Department of Physiology, Pomeranian Medical University, 70-111 Szczecin, Poland; esterabakinowska@gmail.com (E.B.); olejnikjoanna25@gmail.com (J.O.-W.); kajetan.kielbowski@onet.pl (K.K.); skoryk.anastasiiarivne@gmail.com (A.S.); 2 Independent Laboratory of Community Nursing, Pomeranian Medical University, 71-210 Szczecin, Poland

**Keywords:** chronic kidney disease, sarcopenia, inflammation, metabolic dysregulation, gut dysbiosis, microRNA

## Abstract

Chronic kidney disease (CKD) is a progressive disorder associated with a decline in kidney function. Consequently, patients with advanced stages of CKD require renal replacement therapies, such as dialysis and kidney transplantation. Various conditions lead to the development of CKD, including diabetes mellitus, hypertension, and glomerulonephritis, among others. The disease is associated with metabolic and hormonal dysregulation, including uraemia and hyperparathyroidism, as well as with low-grade systemic inflammation. Altered homeostasis increases the risk of developing severe comorbidities, such as cardiovascular diseases or sarcopenia, which increase mortality. Sarcopenia is defined as a progressive decline in muscle mass and function. However, the precise mechanisms that link CKD and the development of sarcopenia are poorly understood. Knowledge about these linking mechanisms might lead to the introduction of precise treatment strategies that could prevent muscle wasting. This review discusses inflammatory mediators, metabolic and hormonal dysregulation, gut microbiota dysbiosis, and non-coding RNA alterations that could link CKD and sarcopenia.

## 1. Introduction

Chronic kidney disease (CKD) is a persistent disorder characterised by structural abnormalities or a gradual decline in kidney function due to kidney damage lasting for a minimum of 3 months, with associated health consequences. According to the current classification system for chronic kidney disease used by Kidney Disease Improving Global Outcomes (KDIGO), kidney disease is categorised based on its cause, the glomerular filtration rate (GFR) (G1–G5), and the level of albuminuria (A1–A3). This classification system is abbreviated as CGA [1,2]. The most common causes of CKD include diabetes (mostly type 2), hypertension, Alport disease, chronic glomerulonephritis, polycystic kidney disease, chronic pyelonephritis, chronic use of anti-inflammatory medications (non-steroidal anti-inflammatory drugs (NSAIDs) such as ibuprofen and diclofenac), congenital malformations, autoimmune diseases, and prolonged acute kidney disease [3,4]. CKD has a relatively high prevalence, affecting approximately 10% of the population worldwide [5]. It cannot be reversed and gradually worsens over time. Additionally, it is linked to an increased risk of cardiovascular problems. The characteristics associated with declining kidney function manifest during more advanced stages of CKD [3,6]. There are various strategies to reduce the probability of kidney failure in individuals with CKD. These measures consist of regulating blood pressure, addressing metabolic acidosis (MA) and preventing protein breakdown, refraining from smoking, adjusting diet to limit protein consumption, managing diabetes appropriately, and administering angiotensin-converting enzyme inhibitors or angiotensin receptor blockers for patients with proteinuria exceeding 500 mg per 24 h. While these actions may mitigate the symptoms, they do not eliminate CKD [3,7].

Individuals with CKD are at an increased risk of developing several conditions such as cardiovascular illnesses, sarcopenia, and cognitive dysfunction, among other clinical outcomes [8,9]. As individuals age, their GFR decreases, leading to an increased risk of complications associated with kidney failure [9]. Studies suggest that the lifespan of individuals with kidney problems is impacted by their age and GFR [6]. Patients with an estimated glomerular filtration rate (eGFR) of 60 mL/min/1.73 m^2^ have a life expectancy of 30.5–34.6 years. With an eGFR of 45–59 mL/min/1.73 m^2^, life expectancy decreases to 24.5–28.7 years. Furthermore, with an eGFR of 30–44 and 15–29 mL/min/1.73 m^2^, life expectancy is further reduced to 14.5–16.5 and 9.1–10.4 years, respectively [6]. Women have a longer life expectancy than men at all ages and eGFR categories, except for eGFR 15–29 mL/min/1.73 m^2^, where there is no gender difference in life expectancy [6]. The predominant CKD treatment modalities involve replacement therapies such as haemodialysis, peritoneal dialysis, and kidney transplantation [3].

CKD is associated with metabolic alterations, including dyslipidaemia, oxidative stress, thyroid dysfunction, MA, accumulation of uraemic toxins, inflammation, calcium-phosphate metabolism disorders, and vitamin D deficiency, among others. Chronic inflammation, characterised by increased levels of tumour necrosis factor α (TNF-α), interleukins (e.g., IL-6 and IL-8), interferon γ (IFN-γ), and cytokines that signal through nuclear factor kappa B (NF-κB), can result in hormonal imbalances and metabolic disturbances, such as protein breakdown and the development of metabolic syndrome. It is necessary to have a comprehensive understanding of the processes that contribute to abnormalities, including sarcopenia, to prevent them effectively [10]. 

Sarcopenia is defined as a progressive decline in muscle mass and function, leading to a notable decrease in overall well-being. In a large meta-analysis that included 42,041 patients with CKD (stages 3a–5), the prevalence of sarcopenia was 24.5% [11]. Moreover, it represents a significant burden in the pediatric CKD population [12]. Studies have indicated that individuals diagnosed with sarcopenia encounter difficulties in performing everyday tasks. Additionally, they tend to experience elevated levels of anxiety and sadness [13]. In elderly patients with CKD, sarcopenia is associated with an elevated risk of frailty [14]. Moreover, the concomitant presence of CKD and sarcopenia is associated with elevated mortality [15]. Sarcopenia is an increasingly prevalent global health concern. To provide personalised treatment, it is necessary to assess and address nutritional deficiencies and the exercise capacity of the patient [16]. This review discusses the potential mechanisms that contribute to the pathogenesis of CKD-related sarcopenia, including inflammation, metabolic and hormonal dysregulation, gut dysbiosis, and altered expression of microRNAs (miRNAs).

## 2. Mechanisms Linking Chronic Kidney Disease and Sarcopenia

### 2.1. Inflammation

CKD is strongly associated with inflammation. Specifically, the systemic inflammatory response index (SIRI), which is composed of lymphocyte, neutrophil, and monocyte counts, has been correlated positively with the prevalence of CKD [17]. Similarly, a link between the disease and the systemic immune–inflammation index (SII, which includes lymphocyte, neutrophil, and platelet counts) has been observed [18]. Systemic inflammation in patients with CKD can also be detected by monitoring inflammatory markers, such as C-reactive protein (CRP), the levels of which are elevated in patients with renal impairment [19,20]. Importantly, patients with CKD have higher levels of circulating pro-inflammatory mediators, including IL-6 and TNF-α [21,22], and there is higher expression of their receptors [23,24].

The altered inflammatory profile in patients with CKD could contribute to the development of muscle wasting and sarcopenia. Pro-inflammatory mediators may enhance muscle protein degradation and stimulate the expression of inhibitors of muscle growth. Analyses of muscle biopsies from patients with CKD have demonstrated elevated expression of Toll-like receptor 4 (TLR4), TNF-α, and elements related to NF-κB, a major regulator of inflammation [25]. A large meta-analysis demonstrated negative correlations between CRP, IL-6, and TNF-α levels and muscle strength [26]. When comparing patients with CKD and sarcopenia with patients with CKD but without sarcopenia, the former group was characterised by elevated levels of CRP [27]. This observation may indicate that CRP could also actively participate in the process of muscle wasting. Interestingly, exposure of myotubes to CRP decreases muscle cell diameters and reduces synthesis of muscle proteins. Mechanistically, CRP suppresses phosphorylation of AKT and enhances AMP-activated protein kinase (AMPK) phosphorylation [28]. However, the impact of AMPK on muscle cell functionality appears to be multidirectional. In the above-mentioned study, CRP enhanced AMPK phosphorylation, which accompanied reduced muscle protein production and decreased cell size. Similarly, in an early study published in 2002, the use of an AMPK agonist decreased muscle protein synthesis in rats [29]. By contrast, activation of AMPK signalling has been suggested to improve sarcopenia due to its role in regulating mitochondrial functionality [30,31,32]. Apart from AKT and AMPK, CRP affects other signalling pathways. Specifically, it increases phosphorylation of extracellular signal-regulated kinase (ERK)1/2 and c-Jun N-terminal kinase (JNK), which belong to the mitogen-activated protein kinase (MAPK) pathway [33]. MAPK regulates several major cellular mechanisms, including myogenesis. For example, ERK1/2 is considered as a repressor of muscle differentiation [34]. On the other hand, Wang et al. [35] showed that mesenchymal stem cells improved muscle functionality in an in vivo model of dexamethasone-induced muscle atrophy. Mechanistically, these stem cells could stimulate ERK1/2 signalling. Given that there are numerous regulators and upstream mediators of MAPK signalling, studies need to precisely analyse the influence of these regulators on MAPK activity before MAPK can be used as a therapeutic target.

In contrast to CRP levels, researchers have reported no significant difference in IL-6 levels between patients with CKD with and without sarcopenia [27,36]. However, when analysing non-CKD-related sarcopenia, researchers have noted higher IL-6 levels [37]. The ability of IL-6 to contribute to muscle protein breakdown was first demonstrated decades ago in an in vivo experiment [38]. Moreover, early studies showed that blocking the activity of IL-6 could reduce muscle protein degradation in animal oncological models [39,40]. Nevertheless, the clinical significance of IL-6 and its relationship with CKD needs to be established. In an important study, Raj et al. [41] found that haemodialysis was associated with an elevated release of IL-6 by the muscles due to muscle catabolism. Therefore, the authors suggested that haemodialysis enhances the secretion of IL-6, which can act as exocrine signal for other tissues or participate in skeletal muscle alterations. IL-6 acts via classic and trans-signalling mechanisms. Briefly, it can bind to its membrane-bound receptor and form a complex that then interacts with gp130. Moreover, IL-6 can bind to a soluble receptor that is secreted by other cells. Subsequently, the complex can bind to the membrane-bound gp130. This binding leads to activation of the Janus kinase (JAK)/signal transducer and activator of transcription (STAT) axis [42,43]. Stimulation of muscle cells with IL-6 enhances STAT3 phosphorylation, which is accompanied by the induction of atrophy [44]. Furthermore, transfection of myofibers with constitutively activated STAT3 could decrease the fibre diameter [45]. Therefore, it is worth asking whether targeting the JAK/STAT pathway could suppress sarcopenia. Consistently, several studies have suggested positive effects of JAK/STAT inhibitors on muscle functionality [45,46,47]. Interestingly, phosphorylation of STAT3 is also regulated by a non-receptor tyrosine kinase Fyn, and knockdown of Fyn could protect against the development of sarcopenia [48]. Furthermore, CKD has been associated with muscle senescence, another process that links inflammation and muscle wasting [49].

**Figure 1:**
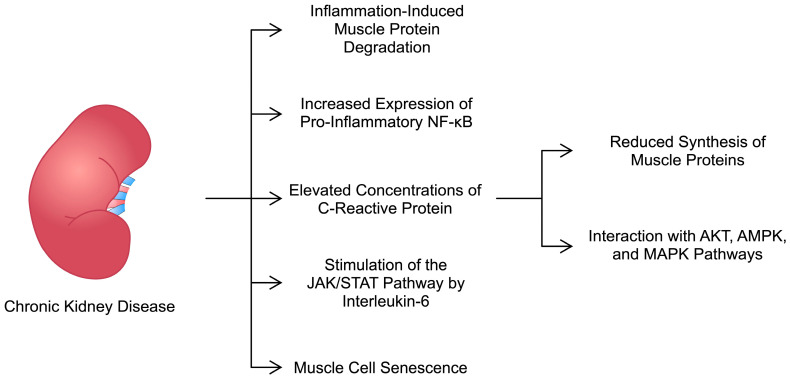
Hypothetical processes induced by chronic kidney disease that could promote the development of muscle wasting and sarcopenia. AMPK—AMP-activated protein kinase; JAK/STAT—Janus kinase/signal transducer and activator of transcription; MAPK—mitogen activated protein kinase; NF-κB—nuclear factor kappa B.

Overall, inflammatory mediators are suggested to participate in several mechanisms that could lead to the development of sarcopenia (Figure 1). However, the precise pathways and contributions remain unknown. In an in vitro study, researchers found that muscle cells collected from patients with CKD demonstrated features of cachexia when cultured. Specifically, they showed enhanced protein degradation compared with cells from controls. Nevertheless, the expression of inflammatory mediators did not differ between both groups [50]. Therefore, it should be explored whether CKD affects the activity of pro-inflammatory cytokines or whether these molecules are involved in an early process of muscle wasting. The conflicting results reported in the literature could be the result of using different models of muscle atrophy, selecting different study populations (pre-dialysis vs. dialysis or different stage of CKD), and the length/chronicity of the disease, which could impact the state of inflammation. 

### 2.2. Metabolic and Hormonal Dysregulation

Chronic renal illness is associated with several disturbances in metabolism and hormonal control—including MA, uraemia, and increased levels of parathyroid hormone (PTH) and insulin-like growth factor (IGF)—which affect muscle functionality and may influence the development of sarcopenia. The accumulation of uraemic toxins and MA lead to insulin resistance and poor muscle mitochondrial energetics. Uraemic toxins build up in the muscle, disrupting mitochondrial enzymes and respiration. Mitochondrial alterations, including changes in their quantity, quality, and oxidative capacity, have a role in the development of mobility problems in individuals with CKD. The primary factors that significantly influence muscle mitochondrial activity are kidney function, inflammation, and oxidative stress. In CKD, muscle mitochondrial activity is mostly determined by MA. Metabolomics has revealed abnormalities in pathways associated with mitochondrial energy metabolism and acid–base balance, which are responsible for insulin resistance in CKD [51]. Restoring metabolic homeostasis enhances muscle mass and functionality, leading to reduced mobility limitations and a lower risk of self-harm [52].

MA occurs in CKD when the kidneys are unable to eliminate the acid load, resulting in a surplus of hydrogen ions (H^+^) and a low concentration of tCO_2_ [53]. It has a negative impact on calcium and phosphate metabolism in individuals with CKD. Furthermore, it reduces the responsiveness of the calcium receptor and increases the release of PTH. MA can elevate β2-microglobulin blood levels, a phenomenon that promotes the formation of β2-microglobulin amyloidosis. Research has shown that renal insufficiency triggers the breakdown of skeletal muscle proteins through a process called acidification-dependent ubiquitination [54,55]. According to this notion, MA is one of the multiple factors that contribute to a negative protein balance due to inadequate protein synthesis and breakdown [56,57]. The depletion of muscle mass leads to a diminished quality of life, increased risk of hospitalisation, and an elevated mortality rate in patients with kidney failure who are receiving haemodialysis. The decline in normalised protein nitrogen appearance that accompanies the correction of MA demonstrates that correcting this metabolic dysregulation can restore protein balance. In one study, there was no connection between correcting MA and dietary protein consumption. However, the decrease in urea levels could potentially be attributed to a reduction in protein breakdown [52]. Significant advances have been made in the treatment of MA in patients with CKD. For example, administration of oral bicarbonate has been found to elevate serum albumin levels and reduce protein catabolism [58,59,60]. Furthermore, MA triggers the release of prostaglandins by osteoblasts and activates osteoclasts while decreasing osteoblast activity. Therefore, it exacerbates osteodystrophy, a condition characterised by increased bone turnover, and increases the risk of developing osteoporosis. Consequently, it has a significant influence on quality of life and can lead to mobility issues. In kidney transplant recipients, the administration of potassium citrate normalised low tCO_2_ levels and improved bone histology and turnover markers compared to the use of potassium chloride over a period of 1 year. Additionally, in individuals with end-stage kidney disease, the correction of acidosis reduced protein breakdown and improved bone histology [54].

Due to declining renal function in patients with CKD, which is accompanied by enzymatic activity in the liver and dysbiotic flora, elimination of uremic toxins is reduced. Protein-bound uraemic toxins that accumulate in patients with CKD exhibit various biological activities and dysregulate molecular pathways. They are particularly detrimental for individuals who follow a high-protein diet. Research indicates that a diet high in protein increases the concentration of uraemic toxins, which in turn decreases the endurance for physical exercise in mice with CKD [61]. During CKD progression, there is a gradual buildup of protein-bound uraemic toxins, such as indoxyl sulfate (IS), *p*-cresyl glucuronide (pCG), *p*-cresyl sulfate (PCS), and indole-3-acetic acid. These compounds are produced from aromatic amino acids, namely, tryptophan (Trp), tyrosine, and phenylalanine, and through the metabolic activity of the gut microbiota. IS, which is part of the aforementioned group, is eliminated by the proximal tubules of the kidneys in healthy individuals. However, in patients with renal impairment, it accumulates due to a likely decrease in cellular transport facilitated by the organic anion transporters OAT1 and OAT3. This accumulation can potentially cause additional damage to the tubules and contribute to the progression of CKD. All of the aforementioned factors lead to a decline in bone production due to the activation of oxidative stress in osteoblasts and the production of PTH, ultimately promoting the formation of adynamic bone [62]. The uraemic environment may induce malfunction in myoblasts by generating oxidative stress and altering mitochondrial activity [63]. In C2C12 cells, a myoblast cell line, IS, enhanced the production of substances associated with the degradation of skeletal muscle, reactive oxygen species (ROS), and inflammatory cytokines (TNF-α, IL-6, and transforming growth factor beta1 [TGF-β1]) [64]. IS hinders the growth of myoblasts and triggers the development of atrogin 1. It also reduces the activity of promyogenic AKT signalling and the expression of myoD, leading to altered myomodulation [65]. Accumulating uremic solutes can stimulate the activity of signaling pathways associated with inflammatory responses, such as MAPK or NF-κB. These mechanisms are associated with pro-inflammatory phenotypes of cells, which highlights the influence of uremia on different tissues [66]. According to the study by Stockler-Pinto et al., the authors observed that in patients undergoing hemodialysis, uremic toxin concentrations are positively correlated with the expression of NF-κB. Simultaneously, levels of IS are also positively associated with that of C-reactive protein (CRP). Conversely, uremic toxins are negatively correlated with the expression of nuclear E2-related factor 2 (Nrf2) [67]. It is an antioxidative molecule, and its deficiency has recently been discussed in the context of CKD and muscle wasting [68].

The parathyroid glands secrete PTH, a polypeptide that plays a crucial role in regulating extracellular calcium levels. PTH production increases serum calcium levels through its effects on the bones and kidneys. PTH-induced stimulation of osteoblasts in bone results in an increase in both the quantity and activity of osteoclasts. PTH suppresses activity in the renal distal tubules [69]. The process of phosphate reabsorption is inhibited by blocking a sodium-dependent phosphate co-transporter, which in turn promotes calcium resorption through an electrochemical gradient. Furthermore, PTH indirectly elevates serum calcium levels by promoting the synthesis of 1-hydroxylase in the proximal tubules. This, in turn, enhances the generation of active vitamin D, a sterol hormone that functions in the intestine to enhance calcium absorption [69]. Regulating PTH levels is a major therapeutic tool in manipulating bone health and thereby influencing linear growth; however, optimal PTH levels throughout the course of CKD have not been well established [69]. Kir et al. examined the role of PTH in a mouse model with muscle atrophy. PTH activated thermogenic genes, including UCPI, in mice with cachexia after nephrectomy. Atrogin-1, muscle-specific RING finger protein 1 (MuRF1), and myostatin gene expression were elevated in muscle tissue, while IGF-1 gene expression was reduced. Additionally, the authors demonstrated that the absence of PTH receptors in adipose tissue hindered the increase in thermogenic gene expression and averted muscle atrophy [61,70]. The loss of skeletal muscle mass occurred alongside a reduction in the expression of the growth-promoting hormone IGF1 and the activation of genes associated with muscle atrophy, namely, MuRF1, atrogin-1, and myostatin [70]. Alterations in the IGF pathway are important in the pathogenesis of sarcopenia. This dysregulation is marked by deviations in growth hormone (GH) and IGF-1 signal transduction, along with interactions with other molecules [71]. Typically, the pathway that incorporates insulin/IGF-1/phosphatidylinositol 3-kinase (PI3K) and AKT enhances muscle protein synthesis through mammalian target of rapamycin (mTOR). Additionally, protein degradation is reduced by inhibiting the FoxO pathway. Reduced Akt activity leads to the activation of FoxO transcription factors and atrogen-1. The process of muscle wasting begins when there is a reduced insulin response and increased atrogin-1 activity [61].

Thyroid hormones play a vital role in muscle growth, regulating the cycles of muscle contraction and relaxation, providing energy, maintaining glucose balance, and repairing muscle injury. Optimal intracellular triiodothyronine (T3) levels play a vital role in promoting muscle growth and maintaining mitochondrial function in smooth muscle cells. Patients with CKD can greatly benefit from assessing their thyroid hormone levels and evaluating the impact of hormone replacement on muscle loss [72].

Vitamin D deficiency is common in patients with CKD and has a significant impact on musculoskeletal conditions. Vitamin D supplementation may enhance physical performance [3]. Nevertheless, the efficacy and potential risks of administering vitamin D compounds to patients with CKD remain uncertain. These risks include hypercalcemia and hyperphosphatemia [73]. Thus, although vitamin D deficiency may play a role in muscular dysfunction in CKD, the administration of vitamin D compounds should be approached with caution.

### 2.3. Gut Microbiota Dysbiosis

Immunosenescence refers to changes in the immune system associated with ageing. Due to the weakened immune profile, there is a link between immunosenescence and CKD [74,75]. The human gut microbiota comprises over 100 trillion bacteria [76]. These microorganisms collectively encode at least 150 times more genes than the human genome, collectively referred to as the microbiome [77]. The two dominant bacterial phyla of the gut microbiota are *Firmicutes* and *Bacteroidetes*. Disruption of the gut microbiota correlates with inflammation and a heightened cardiovascular risk and can lead to metabolic disorders, consequently resulting in progressive loss of proper kidney function. Over the past few years, an increasing body of research has highlighted the potential efficacy of ameliorating gut microbiota dysbiosis to improve CKD symptoms, including proteinuria [78].

Gut microbiota dysbiosis is characterised by an imbalance in microbial composition and can be exacerbated by various factors, including the use of antibiotics and other medications; diets that lack sufficient fibre and are rich in ultra-processed foods; low intake of whole grains, fruits, and vegetables; excessive consumption of fats and salt; increased consumption of red meat; excessive sanitation practices; and delivery via caesarean section. Such influences collectively contribute to the disruption of the gut microbiota, which in turn may play a significant role in the development and progression of CKD [79]. A high-salt diet may influence the gut microbiota in a manner that could interact with the progression of CKD. A decrease in several species of bacteria, including those from the genus *Lactobacillus*, has been observed in mice fed a high-salt diet [80]. Therefore, a vicious cycle occurs: elevated urea levels during CKD lead to alterations in the gut microbiota, which may increase the synthesis of intestinal toxins and affect the permeability of the intestinal epithelial barrier. Enterocytes and the outer mucin layer form a complex multilayered structure, facilitating intricate bidirectional metabolic and immunological interactions. These changes may accelerate the progression of kidney damage [81].

#### 2.3.1. Metabolites of the Gut Microbiome and Their Impact on CKD and Sarcopenia

Various metabolites produced by the gut microbiota induce metabolic changes in the intestines and other organs, such as the muscles and kidneys. Exposure of the gut microbiota to urea via gastrointestinal secretion triggers the conversion of urea into ammonia by bacterial urease. An elevated urea concentration leads to the proliferation of bacterial families containing urease. Patients with end-stage kidney disease maintained on haemodialysis exhibit an expansion of bacterial families producing uricase, as well as enzymes producing indole and *p*-cresol, compared with healthy individuals [82]. Exposure of colonic epithelial cells to urea partially affects tight junctions, facilitating the translocation of endotoxins and microbial fragments and leading to inflammation that increases epithelial barrier disruption [83]. Oedema of the intestinal wall, along with intermittent hypotension during haemodialysis, worsens the impairment of the colonic epithelial barrier [84]. Ammonia and ammonium hydroxide arising from urea metabolism play a pivotal role in compromising the intestinal epithelial barrier. In patients with CKD and sarcopenia, this breach of the intestinal epithelial barrier facilitates the absorption of microbial toxins, triggering systemic inflammation, which can contribute to cardiovascular diseases, anaemia, and protein wasting [82]. 

The uraemic toxin IS, a gut microbiota metabolite, is a potential factor that induces intestinal barrier injury by inhibiting the mitophagic flux of intestinal epithelial cells (IECs) associated with CKD. In mice and patients with CKD, IS-induced repression of dynamin-related protein 1 (DRP1) has been observed through the transcription factor interferon regulatory factor 1 (IRF1), which possesses a C-terminal repression domain that negatively regulates the transcription of target genes [85]. The IRF–DRP1 axis may form a feedback loop regulating mitochondrial function. The observation of increased IRF1 expression and its suppression of DRP1 may serve as a significant focal point for further research. Furthermore, IS is an endogenous agonist for the aryl hydrocarbon receptor (AhR) in the circulation, exerting pro-fibrotic and pro-inflammatory effects partly through activation of the AhR/NF-κB pathway [86]. Given the increasing evidence of the interrelationships between CKD and the gut microbiota, the link between CKD and sarcopenia is also drawing attention. So-called uraemic dysbiosis may become a new therapeutic target for patients with CKD and sarcopenia. Studies have shown a direct link between IS and metabolic changes in skeletal muscle, leading to uraemic sarcopenia in CKD. This connection underscores the role of IS in contributing to muscle atrophy and weakness observed in patients with CKD [87]. 

Metabolomic studies have shown that in muscles, IS enhances the antioxidant response through the activation of nuclear factor erythroid 2-related factor 2 (NRF2). This overactivation of the antioxidant response leads to a reduction in the tricarboxylic acid (TCA) cycle, resulting in insufficient adenosine triphosphate (ATP) production, which causes muscle weakness and sarcopenia. Due to their high metabolic activity, muscle cells are particularly susceptible to mitochondrial dysfunction and degradation, making them especially vulnerable to IS toxicity. The changes induced by IS may represent a potential therapeutic target for sarcopenia in the future [87,88]. Another metabolite produced by the gut microbiota is Trp, an amino acid with an indole structure. Its metabolites can exacerbate intestinal inflammation and, consequently, promote the development of sarcopenia and CKD. Moreover, an increasing body of research suggests the potential use of Trp metabolites, such as kynurenine (Kyn), 5-hydroxyindoleacetic acid (5-HIAA), and indole, as biomarkers for various diseases. The gut microbiota can metabolise indole derivatives produced from Trp, which can act as ligands for the AhR. Increased activation of AhR can lead to mitochondrial damage, resulting in muscle damage and atrophy [89,90,91]. The gut microbiota can enhance the activity of the enzyme indoleamine 2,3-dioxygenase 1 (IDO1), which is activated during inflammation. This leads to the depletion of Trp and the accumulation of its metabolites, Kyn, 5-HT, and indole, further fuelling the inflammatory state [92].

#### 2.3.2. Effects of Specific Bacteria on the Development of CKD and Sarcopenia

CKD can lead to sarcopenia, which is associated with a high risk of mortality [93]. Similarly to CKD, changes in the colonisation of the bacterial families *Prevotellaceae* and *Lactobacillaceae* have been observed in cases of sarcopenia. Biotics appear to offer promise in regulating the pathologically altered gut microbiome. A rat model yielded satisfactory results with supplementation of *Lactobacillus* spp., with increased intestinal barrier integrity and a reduction in cachexia and sarcopenia [94]. Similarly, the growth of *Bifidobacterium* can positively impact the gut microbiome in the settings of both CKD and sarcopenia [94,95]. In another study, elderly patients who received 1-kestose supplementation for 12 weeks showed an increase in *Bifidobacterium longum*, which was associated with a reduction in the severity of sarcopenia [96]. 

**Figure 2:**
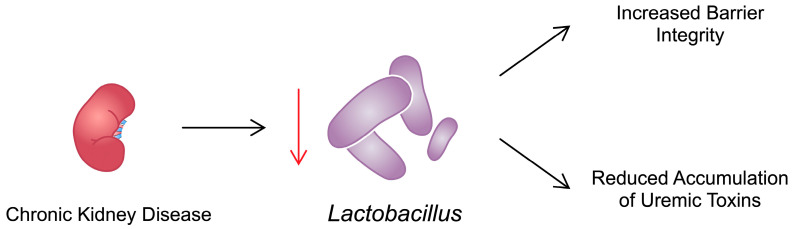
The state of CKD reduces the presence of *Lactobacillus*, which is associated with suppression of their beneficial effects that are associated with muscle health.

A growing number of studies have focused on the effects of the biotic supplement AST-120, which positively affects the composition of the gut microbiota (primarily *Lactobacillus*) in CKD [97]. AST-120 exerts its beneficial effects by preventing accumulation in the circulation of uraemic toxins such as IS and PCS due to its effect on the gut microbiota. Additionally, AST-120 has been shown to alleviate muscle atrophy, enhance exercise capacity, and promote mitochondrial biogenesis [98]. RECOVERY, a multicentre randomised clinical trial, investigated the influence of AST-120 on muscle functionality in patients with CKD. AST-120 did not improve gait speed or hand grip strength. However, the use of IS adsorbent reduced the number of patients with sarcopenia and low muscle mass to the point where there was no significant difference between the study and control groups [99]. In the RECOVERY trial, an analysis of IS levels demonstrated that patients with higher concentrations of this toxin had lower skeletal muscle indexes and hand grip strength. Moreover, IS levels were negatively correlated with eGFR [100]. In another study by Jerez-Moralez et al., the authors observed that administration of *Lactobacillus bulgaricus* 6c3 Strain to nephrectomized rats for 16 weeks was associated with reduced concentrations of IS [101]. A depiction of the impact of CKD on Lactobacillus and its effect on muscle functionality is presented on Figure 2. Mitochondrial function is impaired in both sarcopenia and CKD, a common link between the conditions. Indeed, numerous studies have demonstrated that CKD is associated with mitochondrial dysfunction and a reduced number of mitochondria in skeletal muscle due to the negative impact of uraemic toxins. These toxins accumulate in the muscles, leading to decreased activity of various mitochondrial enzymes, resulting in insufficient ATP production and, consequently, muscle weakness [51,102].

The increase in urea and uraemic toxins in CKD has a myriad of negative effects. First, there are the aforementioned adverse effects on muscles and their mitochondria. Second, there is the additional disruption of the gut microbiota, which amplifies the production of these toxins. In CKD, the gut microbiota becomes dominated by bacteria that produce uraemic toxins such as IS, PCS, and trimethylamine *N*-oxide (TMAO) via tryptophanase and urease. These bacteria include *Proteobacteria*, *Escherichia coli*, *Enterobacter*, *Acinetobacter*, *Proteus*, and *Clostridium perfringens*. Concurrently, the population of short-chain fatty acid-producing bacteria, such as *Lactobacillus* and *Bifidobacterium*, decreases. The disruption of the gut microbiota and increased production of toxins further impair kidney function, which in turn affects muscle function. This leads to gut–muscle crosstalk and creates a network of negative interactions [103]. Other studies have confirmed elevated levels of IS and phenyl sulfate in the serum and urine of rats with CKD. These studies identified gut microbiota producing indole and phenol, specifically *Clostridium* cluster IV, *Alistipes*, and *Bacteroidia* [82,104]. 

Uchiyama et al. [105] investigated the direct impact of uraemic dysbiosis on the progression of sarcopenia in mice. They demonstrated that uraemia alters the composition of the gut microbiota and the function of the epithelial barrier. In mice with CKD, there was an increase in *Allobaculum* and a decrease in *Lactonifactor*, which may contribute to sarcopenia. Additionally, a decrease in bacteria possessing the butyrate kinase gene, such as members of the families *Lactobacillaceae* and *Prevotellaceae*, has been observed in end-stage kidney disease [82,104]. This reduction may be due to a stringent diet aimed at preventing hyperkalaemia. Patients with this condition present a significant decrease in methanol production, a byproduct of butyrate fermentation [106]. Butyrate, a short-chain fatty acid, plays a crucial role in the proper functioning of colonocytes, including passive diffusion, thereby enabling the activity of ion exchange transporters. Adequate levels of butyrate in the intestine can help prevent the progression of colitis [107]. 

Guo et al. [108] assessed changes in the gut microbiota of elderly individuals with initially low handgrip strength. They demonstrated that the abundance of *Parabacteroides* and *Intestinibacter* was higher in individuals with low handgrip strength. The abundance of these bacteria correlated negatively with levels of cinnamoylglycine and (2E)-5-hydroxyferulic acid, a cinnamic acid derivative. These compounds have positive effects on muscle cell function, proliferation, and differentiation [109]. Sugimura et al. [110] performed a similar study on grip strength and body mass index in a larger study cohort. They found that an increase in the abundance of *Blautia* and *Eggerthella*, along with a simultaneous decrease in *Faecalibacterium*, may mitigate muscle weakness. Picca et al. [111] observed an increase in *Bifidobacteriaceae* and *Eggerthella* and an increase in *Peptostreptococcaceae* and *Dialister* in the group with physical weakness and sarcopenia compared with the control group. Moreover, there was depletion of *Slackia* and *Eubacterium*. They also noted changes in Trp levels, and they occurred earlier compared with the control group. A study conducted on mice showed that colonocytes utilise bacterially produced butyrate as their primary energy source. Butyrate, acting as a histone deacetylase (HDAC) inhibitor, regulates gene expression and serves as the primary energy source for the colonic epithelium. Butyrate oxidation takes place in the mitochondria [112]. 

The low abundance of bacteria such as *Lactobacillus*, *Bifidobacterium*, *Bacteroides*, and *Prevotella* may play a significant role in CKD aetiology [113]. When evaluating a group of 50 patients with liver cirrhosis, including 19 with sarcopenia, researchers also linked changes in the gut microbiota to disease conditions. The gut microbiota of patients with both sarcopenia and liver cirrhosis showed decreased levels of *Prevotella*, *Akkermansia*, and *Methanobrevibacter*, and an increased level of *Eggerthella*, as reported in other studies, compared to patients with liver cirrhosis without sarcopenia. Additionally, *Klebsiella* was elevated. Furthermore, the authors identified correlation networks and clusters of variables associated with sarcopenia, including the *Klebsiella*/ethanol/FGF21/*Eggerthella*/*Prevotella* cluster; a decrease in bacteria such as *Methanobrevibacter*, *Prevotella*, and *Akkermansia*; and increases in *Eggerthella* and *Klebsiella*. These changes establish a metabolic and pro-inflammatory network involving ethanol, TMA, myokines such as FGF21, cytokines, and chemokines, which may ultimately contribute to muscle atrophy. Table 1 summarises potential links between bacteria, CKD and sarcopenia.

#### 2.3.3. The Impact of Diet and Drugs

In CKD, traditional dietary regimens often lead to reduced intake of foods rich in fibre, fruits, vegetables and antioxidants. These substances are essential nutrients for the gut microbiome. Butyrate-producing bacteria require the right environment, and it is these bacteria that are lacking in CKD [126]. There is an increasing emphasis on the importance of a low-protein diet, which increases the abundance of bacteria from the *Lactobacillaceae* and *Bacteroidaceae* families as well as *Streptococcus anginosus* and decreases the abundance of *Roseburia faecis* and *Bacteroides eggerthii* [127]. A study by Chen et al. showed that the use of a low-protein diet supplemented with amino acid ketoanalogues can further delay the progression of kidney disease and reduce mortality [128]. 

Other important factors affecting the gut microbiota are medications and their effects on the metabolism of gut bacteria. Frequently used drugs such as metformin, proton pump inhibitors (PPIs), some antibiotics, and laxatives can negatively alter the gut microbiota. In the case of PPIs, it is suggested that their effects on microflora are due to changes in gastrointestinal pH and direct inhibition of *Dorea* and *Ruminococcus* bacteria. In contrast, antibiotics have been observed to significantly reduce *Bifidobacterium* abundance. In the case of metformin, on the other hand, an enrichment of *E. coli* was observed in faecal samples of users of the drug [129,130,131]. Additionally, in CKD, physical activity plays an important role in the greater diversity of the gut microbiota. Engaging in sports is associated with greater bacterial diversity, which is lacking in CKD. In particular, *Faecalibacterium*, *Lachnospira*, and *Akkermansia*, which are deficient in CKD, respond to increased activity [132]. With the growing popularity of supplementation with various substances, it is worth looking at butyrate supplementation and its effect on microbiota composition. The effective functioning of the butyrate–renal axis significantly reduces renal dysfunction and inhibits vascular calcification and renal fibrosis in CKD [133,134,135,136]. A beneficial effect of supplementation of *Faecalibacterium prausnitzii*, which is a butyrate producer, has been observed in mice with CKD. Supplementation of this bacterium and increased butyrate production contribute to improved intestinal tightness and renal function in CKD [137]. The topic of nutrition and diet in CKD is important and deserves further research and in-depth analysis, as the influences of food, physical activity, and lifestyle significantly affect the development of normal bacterial flora, and thus may limit further progression and complications of CKD [138,139].

### 2.4. microRNA

miRNAs are short molecules that bind to their target messenger RNAs (mRNAs) and suppress translation. Dysregulated miRNAs have been observed in numerous diseases, and these abnormalities could be used in the diagnostic process. Furthermore, changes in miRNAs alter the expression of target genes and can contribute to the pathogenesis of a disease. CKD significantly alters the expression of miRNAs in the kidney [140]. In addition, CKD can change the levels of circulating miRNAs [141]. These alterations might modify the physiology and functionality of muscle cells and contribute to the development of sarcopenia. Furthermore, the chronic metabolic and hormonal impairments observed in CKD may affect the expression of miRNAs directly in skeletal muscles. For example, Wang et al. [142] reported that 12 miRNAs were significantly dysregulated in the muscles of CKD mice. 

In CKD mice, researchers noted a 35% decreased expression of miR-26a. Moreover, treatment of satellite cells with serum from CKD mice also reduced the expression of miR-26a. Downregulation was associated with enhanced presence of the phosphatase and tensin homolog protein (PTEN), a negative regulator of the Akt signalling pathway and a marker of muscle atrophy. Consistently, there was reduced phosphorylation of Akt [143]. The Akt/mTOR signalling pathway is highly associated with muscle cell physiology. Specifically, enhanced activity of the pathway is found in hypertrophic muscles, while muscle atrophy is associated with the downregulated pathway [144]. Zhang et al. [145] reported reduced miR-26a-5p expression in the muscle of unilateral–ureteral obstruction (UUO) mice models. Inducing the expression of miR-26a was associated with suppressed muscle atrophy [143,145]. miR-26a participates in myoblast differentiation, as well as muscle regeneration after injury. Suppressing miR-26a was associated with delayed post-injury regeneration, which was accompanied with dysregulated expression of the miR-26a targets *SMAD1* and *SMAD4* [146]. 

In an early study investigating miR-26a published by Wong and Tellam, the authors demonstrated that overexpression of miR-26a enhanced myogenesis of murine C2C12 myoblasts. Mechanistically, the molecules targeted enhancer of zeste homolog 2 (EZH2), a histone methyltransferase [147]. The expression of EZH2 changes during differentiation of muscle cells, and its activity is associated with repression of muscle gene expression [148]. However, it plays an important role in muscle functionality, as its ablation reduces the number of muscle stem cells, inhibiting the processes of myogenesis and muscle regeneration [149]. Under septic conditions, EZH2 interacts with metastasis-associated lung adenocarcinoma transcript 1 (MALAT1), an oncogenic long non-coding RNA, to promote apoptosis of skeletal cells [150].

miRNAs are known to mediate the expression of numerous targets, thus regulating the activity of several signaling pathways and processes. miR-26a has also been found to target different mRNAs in models and experiments unrelated to sarcopenia. For instance, it has been shown to regulate inflammatory responses in macrophages. Introduction of miR-26a delayed serious outcomes of LPS injection in mice. Mechanistically, miR-26a mimics reduced the expression of cyclooxygenase-2 (COX-2) in peritoneal macrophages [151]. Reducing the expression of COX-2 has been associated with improved inflammatory status of skeletal muscles in naturally ageing mice [152]. In a colitis model, the molecule was found to target TLR3, further confirming its anti-inflammatory effects [153]. We have previously mentioned that the expression of TLR4 is elevated in the skeletal muscles of CKD patients [25]. Thus, interactions with TLRs might represent another potential mechanism to restore altered muscle homeostasis in the state of CKD. Furthermore, Zheng and colleagues showed that miR-26a suppressed the expression of connective tissue growth factor (CTGF) in a model of renal fibrosis [154]. In skeletal muscles, CTGF has been suggested to induce protective effects, as its selective muscle deficiency could features of muscle dystrophy in δ-sarcoglycan–null (Sgcd−/−) mice models [155]. Recently, miR-26-5p was also shown to target the Acyl-CoA synthetase long-chain family member 3 (ACSL3) in adipocytes [156]. 

miR-29 is also altered in the skeletal muscle in the context of CKD. There are three isoforms of this molecule (miR-29a, miR-29b, and miR-29c), and their expression is reduced in the muscles of CKD mice. Mechanistically, reduced miR-29 expression could be associated with increased expression of Yin Yang 1 (YY1), a transcription factor that regulates muscle differentiation. Importantly, introduction of miR-29 has been associated with increased differentiation of C2C12 myoblasts into myotubes [142]. Similarly, there was reduced expression of miR-29a-3p in the muscles of UUO mice. Furthermore, injection of exosomes loaded with miR-29 into the muscles of UUO mice increased their muscle mass. In addition to YY1, miR-29 regulates the expression of pro-fibrotic TGF-β [157]. UUO mice showed increased expression of YY1 and markers of atrophy, including MuRF1 and PTEN. By contrast, the muscle-specific markers myogenin and myoD were downregulated. These alterations could be improved by intramuscular injection of miR-29a. Interestingly, Wang et al. [158] identified a muscle–kidney interaction, as intramuscular injection of miR-29a reduced fibrotic features of UUO mice. Liu et al. [159] reported similar findings: overexpression of the miR-29ab1 cluster protected against muscle atrophy. 

The above studies have demonstrated that miR-29 affects the expression of important genes implicated in muscle development, regeneration, and/or fibrosis. However, the involvement of miR-29 targets in skeletal muscle physiology seems more complex. Deletion of YY1 from the Pax7-positive muscle progenitor cells was associated with rapid postnatal death in mice due to impaired diaphragm function. Furthermore, in other mouse models, YY1 deletion exacerbated dystrophic muscular changes [160]. However, physiologically, the expression of YY1 fluctuates during myocyte differentiation, which suggests that a different YY1 expression profile is required for proper muscle development [161]. Subsequent studies have demonstrated that YY1 suppresses the expression of muscle-specific genes and that regulating YY1 degradation is an important mechanism that mediates myocyte differentiation [162]. Therefore, altered YY1 expression due to dysregulated miRNAs could impair the regenerative properties of muscle cells and may contribute to the development of sarcopenia. miRNAs have been implicated in a broad network of interactions and frequently target and regulate several genes. Consequently, depending on the cellular context, the same miRNAs may regulate different mechanisms. For example, in atrophy models induced by TNF-α, hydrogen peroxide, dexamethasone, and angiotensin II, researchers suggested that miR-29b participates in the pathogenesis of atrophy [163,164]. Furthermore, miR-29 affects dystrophic muscles. Overexpressing the miR-29ab1 cluster in mice was associated with features of Ullrich congenital muscular dystrophy. The authors showed that miR-29 targeted and suppressed the expression of *Col4a1* and *Col4a2* [165]. However, in mdx mice, a model of Duchenne muscular dystrophy, introduction of miR-29 improved muscle physiology [166].

Similarly to miR-26, various target genes have been identified for miR-29 in different disease models. For instance, miR-29b could bind to the 3′UTR region of MMP-2, which was associated with reduced expression of the metalloproteinase [167]. Perhaps this finding may translate into the context of sarcopenia. In an early study by Bar-Shai et al., the authors observed that immobilization of the rat limb for 4 weeks increased the mRNA expression of MMP-2 [168]. Targeting integrin β1 (ITGB1) via miR-29 could also have implications in skeletal muscle health, as ITGB1 mediates satellite cell homeostasis and suppresses dystrophic features [169,170]. Other targets of miR-29 family include laminin C2 [171] and ADAM12-L [172], among others.

**Figure 3:**
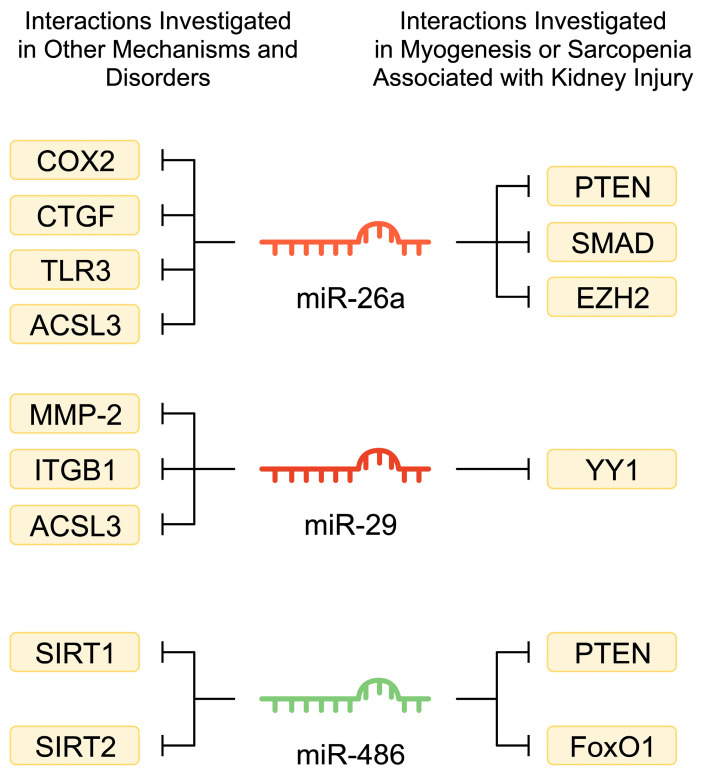
MicroRNA regulate the expression of numerous molecules, mediating the activity of a number of signaling pathways and cellular processes. The figure demonstrates miRNA interactions with target genes that were investigated in the context of myogenesis and chronic kidney disease-associated sarcopenia, as well as selected interactions detected in other disorders.

miR-486 is downregulated in the muscles of CKD mice. Introduction of an miR-486 mimic increased muscle mass and reduced markers of atrophy. Mechanistically, the molecule reduced PTEN and FoxO1 protein expression, suggesting suppression of catabolic responses [173]. As previously mentioned, miRNA molecules can influence various pathways. In certain cases, observed results might seem contradictory and require additional analyses of regulatory mechanisms. miR-486 mediates the expression of sirtuins, a family of proteins that classically are associated with the process of deacetylation. Huang and collaborators reported that miR-486 can target SIRT1 in macrophages, while restoration of SIRT1 expression can suppress inflammatory responses induced by LPS [174]. By stimulating protein homeostasis, suppressing inflammatory responses, and reducing apoptosis, SIRT1 is suggested to induce beneficial effects in sarcopenia, as comprehensively discussed in a recently published review by Yang et al. [175]. In another study, a lucifer reporter assay confirmed that miR-486-3p binds to the SIRT2 mRNA as well [176] (Figure 3).

Exercise is considered to be a method to prevent muscle loss in people with CKD. Intriguingly, exercise has been found to alter the miRNA expression profile in skeletal muscles. Specifically, the expression of miR-23 is reduced in the muscles of CKD mice. Muscle overload increases the expression of miR-23 and miR-27. Importantly, overexpression of these miRNAs significantly improves muscle mass and strength in CKD mice. Furthermore, it suppresses the expression of atrophic markers, myostatin, and caspases [177]. Table 2 summarizes the involvement of the described miRNAs in CKD-associated muscle atrophy. 

Acupuncture and low-frequency electrical stimulation have been shown to counteract muscle atrophy by altering miRNA expression [178,179]. Specifically, miR-181d expression was increased in the skeletal muscles, and there was an increase in miR-181d in circulating exosomes. Interestingly, it was also associated with improved renal blood flow, further confirming the interaction between muscles and kidneys [180]. Other miRNAs could be associated with improved muscle and kidney function. However, there are limited data regarding their role in sarcopenia in the context of CKD. myomiRs, including miR-1, miR-206, miR-208, and miR-133, are miRNAs that are specifically associated with muscle tissue. They have been shown to regulate myogenesis and the stress response [181], and they might link CKD with muscle wasting. 

## 3. Biomarkers

In the previous paragraphs, we aimed to discuss various mechanisms that could be implicated in the pathogenesis of CKD-associated sarcopenia. However, understanding the pathophysiology of the disorder should translate into clinical improvements that would allow for a more rapid diagnosis, identification of markers of treatment response, as well as novel treatment methods. An accumulating number of studies aim to examine potential biomarkers in various fields of medicine. In the context of discussed topic, biomarkers may predict the development of muscle wasting and indicate the severity of muscle atrophy. For instance, markers of renal function may serve as biomarkers of sarcopenia. Specifically, the serum cystatin C to creatinine (SCr) ratio has been suggested as a potential sarcopenic marker. As the SCr level is dependent on muscle mass while cystatin C is not, monitoring these two markers should reflect muscle homeostasis. As demonstrated by An et al., CKD patients with sarcopenia have a significantly increased ratio compared to those with kidney disorder but without sarcopenia. Importantly, the authors also investigated the diagnostic utility of the cystatin C/SCr ratio and demonstrated that the area under the curve (AUC) was 65.6% [182]. Recently, calculation of GFR based on cystatin C has been implemented in the sarcopenia index (SI). In an analysis by Lin et al., the authors used the SI to evaluate its diagnostic potential in examining the presence of sarcopenia in CKD patients. The AUC results for males and females were 64.6% and 75.4%, respectively [183]. In addition, monitoring of myostatin could also be beneficial in evaluating the presence of sarcopenia. Myostatin belongs to the TGF-β superfamily and negatively regulates the muscle marker MyoD, thus negatively regulating muscle mass [184]. Consequently, myostatin inhibitors are being developed and investigated in muscle atrophies and dystrophies [185,186]. Apart from being a therapeutic target, it could be used as a biomarker of sarcopenia. Yasar and collaborators analyzed 130 patients, including those undergoing hemodialysis, peritoneal dialysis, renal transplant recipients, and CKD patients who were not dialysis-dependent. In this cohort, serum levels of myostatin were significantly elevated in patients with sarcopenia [187]. Additionally, monitoring blood levels of elements could also be associated with sarcopenia. For instance, higher manganese levels were observed among hemodialysis patients. Importantly, manganese concentrations were negatively correlated with grip strength [188]. Moreover, Li and colleagues examined the existence of potential correlations between markers of intestinal permeability and skeletal muscle strength in adults. The authors observed a significantly negative association between handgrip strength and diamine oxidase [189]. However, the potential associations between markers of intestinal permeability and sarcopenia and CKD need to be further evaluated. 

## 4. Conclusions and Future Perspectives

Sarcopenia is a dangerous condition in patients with CKD, and it is associated with a worse prognosis. According to the literature, numerous mechanisms seem to be involved in the pathogenesis of CKD-related sarcopenia. In CKD patients, increased expression and concentrations of pro-inflammatory mediators are being observed. In turn, these molecules can contribute to muscle wasting and reduced strength, taking part in the pathogenesis of sarcopenia. Similarly, the chronic state of CKD impairs the ability to eliminate uremic toxins. Consequently, their elevated concentrations contribute to systemic inflammation and oxidative stress together with decreased skeletal muscle. Gut dysbiosis is considered as another mechanism taking part in the progression of CKD. It is an important source of uremic toxins; thus, it might be indirectly associated with muscle health. In addition, CKD is associated with altered expression of miRNA, molecules that significantly regulate gene expression. As miRNAs target numerous genes and mediate the activity of several signaling pathways, their altered expression has been implicated in the development of muscle abnormalities. Despite the abundance of studies investigating the above-mentioned processes, the precise molecular pathways and interactions leading to muscle wasting have not been explored sufficiently. Detailed knowledge about the pathways and cells that participate in the progression of sarcopenia might result in the introduction of precise therapeutics that could eventually improve the quality of life and prognosis of patients with CKD. Future studies should explore the pathways that are involved in muscle degeneration in patients with end-stage kidney disease in greater depth. Furthermore, investigators should examine the impacts of different types of renal replacement therapies on muscle mass and functionality. Interestingly, short-daily haemodialysis has been suggested to improve muscle function compared with conventional haemodialysis [190], which highlights the importance of studying the influence of different dialysis modalities on muscles. Moreover, epigenetics has also been suggested to be involved in the process of muscle wasting [191]; thus, other mechanisms that have been discussed in this review should be explored. Recent studies suggest a deeper association between kidney and muscle, as sarcopenia can also enhance the risk of developing CKD [192]. Furthermore, the combined presence of sarcopenia and low bone mineral density further increases the risk of CKD occurrence [193].

## Figures and Tables

**Table 1:** Summary of potential links between dysregulation of selected pathogens and sarcopenia.

Pathogen/Family	Effect of CKD/Association with Kidney Function	Potential Link with Sarcopenia	References
*Lactobacillaceae*	Reduction in the number of families with enzymes that form short-chain fatty acids (butyrate) in the intestine in CKD	*Lactobacillus pluralis* and *Lactobacillus* spp. supplementation have a positive effect on muscle atrophy	[83,84,94,95,98,113,114]
*Prevotellaceae*	Reduction in the number of families with enzymes that form short-chain fatty acids (butyrate) in the intestine in CKD	Reduced *Prevotellaceae* abundance in Chinese women Reduced *Prevotellaceae* abundance in older people with low muscle strength	[83,84,115,116,117]
*Bifidobacterium*	Decrease in CKD	In a rat model, *Bifidobacterium* supplementation had a positive effect on muscle atrophy Increase in people with physical weakness and sarcopenia	[94,95,96,111,114,116]
*Proteobacteria*	Increase in CKD	Indirect association in an alcohol-abusing group with lower muscle mass based on handgrip strength: increase in *Proteobacteria* Mice treated with metronidazole showed an increase in *Proteobacteria* abundance and a decrease in lower limb muscle mass	[114,118,119]
*Escherichia coli*	Increase in *E. coli* population and enhanced production of indole.	Enhanced *E. coli* growth shown in patients with cirrhosis and muscular atrophy	[113,114,120]
*Allobaculum*	Increase in CKD	Increased *Allobaculum* abundance may contribute to a sarcopenic state	[105]
*Lactonifactor*	Decrease in CKD	Decreased *Lactonifactor* abundance may contribute to a sarcopenic state	[105]
*Alistipes*	Increase (production of indole, a precursor of IS, from tryptophan)	Decreased *Alistipes shahii* abundance in sarcopenia	[82,104,116,121]
*Eggerthella*	Increase in CKD *Eggerthella lenta*	Increased *Eggerthella* abundance	[110,111,116]
*Blautia*	Decrease in CKD	Increased *Blautia* abundance	[110,122]
*Faecalibacterium*	Decrease in CKD	Decreased *Faecalibacterium prausnitzii* abundance in sarcopenia	[110,121]
*Peptostreptococcus*	Growth of *Peptostreptococcaceae* with administration of capsules with a mixture of live bacteria *Enterococcus faecalis*, *Bifdobacterium longum,* and *Lactobacillus acidophilus*) in hemodialysis patients	Increased *Peptostreptococcus* abundance in individuals with physical weakness and sarcopenia	[111,123]
*Dialister*	*Dialister* abundance decreased with increasing severity of CKD	Increased *Dialister* abundance in individuals with physical weakness and sarcopenia	[111,124]
*Pyramidobacter*	CKD+AST-120 group showed significant enrichment in *Pyramidobacter* compared to CKD group without AST-120 treatment	Increased *Pyramidobacter* abundance in individuals with physical weakness and sarcopenia	[111,125]
*Eubacterium*	CKD+AST-120 group showed significant enrichment in *Eubacterium nodatum* compared to CKD group without AST-120 treatment	Decreased *Eubacterium* abundance in individuals with physical weakness and sarcopenia	[111,125]

**Table 2:** Summary of microRNAs with altered expression in chronic kidney disease that mediate muscle functionality.

MicroRNA	Expression in Chronic Kidney Disease Conditions	Mechanism Linking Chronic Kidney Disease and Muscle Functionality	References
miR-26a	↓	Overexpression of miR-26a inhibits muscle atrophy.	[143,145]
miR-29	↓	Overexpression of miR-29 improves C2C12 myoblasts differentiation. Intramuscular injection of miR-29a could reduce muscular atrophy markers.	[142,158]
miR-486	↓	Administration of miR-486 mimic into the muscles of CKD mice increased muscle mass and reduced the expression of atrophy markers.	[173]
miR-23	↓	Overexpression of miR-23 together with miR-27 increased muscle mass and strength.	[177]

↓—decreased; CKD—chronic kidney disease.
